# Changes in numbers needed to treat and hospital care expenditures of optimized indications for primary prevention implantable cardioverter defibrillators: a scenario analysis

**DOI:** 10.1007/s00392-025-02687-4

**Published:** 2025-06-10

**Authors:** M. van Barreveld, P. F. H. M. van Dessel, E. Buskens, L. V. A. Boersma, P. P. H. M. Delnoy, A. E. Tuinenburg, D. A. M. J. Theuns, P. H. van der Voort, G. P. Kimman, T. E. Verstraelen, A. H. Zwinderman, A. A. M. Wilde, M. G. W. Dijkgraaf

**Affiliations:** 1 Amsterdam UMC Location University of Amsterdam, Cardiology, Meibergdreef 9, 1105 AZ Amsterdam, Netherlands; 2 Amsterdam UMC Location University of Amsterdam, Epidemiology and Datascience, Meibergdreef 9, Amsterdam, Netherlands; 3 Medisch Spectrum Twente, Enschede, The Netherlands; 4 Department of Epidemiology, University Medical Centre Groningen, Groningen, The Netherlands; 5 Cardiology Department, St. Antonius Ziekenhuis, Nieuwegein, The Netherlands; 6 Department of Cardiology, Isala Klinieken, Zwolle, The Netherlands; 7 Department of Cardiology, Division of Heart and Lungs, University Medical Centre, Utrecht, The Netherlands; 8 Department of Cardiology, Erasmus MC, Rotterdam, The Netherlands; 9 Department of Cardiology, Catharina Ziekenhuis Eindhoven, Eindhoven, The Netherlands; 10 Department of Cardiology, Noordwest Ziekenhuisgroep, Alkmaar, The Netherlands; 11 Methodology, Amsterdam Public Health, Amsterdam, The Netherlands

**Keywords:** Implantable cardioverter defibrillator, Nationwide registry, Number needed to treat, Health resources, Health care costs

## Abstract

**Aim:**

A strong need exists to better select patients with reduced left ventricular ejection fraction for primary prevention of sudden cardiac death by ICD implantation. This paper reports on the expected clinical and economic benefits of stricter indication scenarios based on minimum probabilities for patients of experiencing appropriate ICD-therapy and/or maximum risks of dying during the first 2 years following ICD implantation.

**Methods:**

Data on clinical events and hospital care expenditures were gathered for patients in the Dutch DO-IT registry. Registry-based prediction models were used to derive individual prior probabilities. Realistic assumptions were made concerning short-term disease courses and related hospital care in absence of ICD implantation. The potential impact of stricter indication scenarios was assessed with changes in numbers needed to treat (NNT) in subpopulations with (NNT-yes) or without (NNT-no) indication for ICD implantation and with changes in the yearly incidence-based national hospital care budget for ICD-based primary prevention.

**Results:**

The NNT-yes under the existing guidelines equalled 42. Not indicating ICD implantation if prior probabilities of receiving appropriate therapy within 2-year post-implant are < 5% seems a promising cutoff with an NNT-yes of 33, an NNT-no of 246 and a national annual reduction in hospital expenditures for ICD-based primary prevention of €11 million (16.7%).

**Conclusions:**

Stricter indication criteria for primary prevention ICD implantation enable the selection of patient subpopulations with high numbers needed to treat, in which unnecessary harm can be forgone and substantial savings can be accomplished. The scenario analysis facilitates rationing of indication policies for ICD implantations.

**Graphical abstract:**

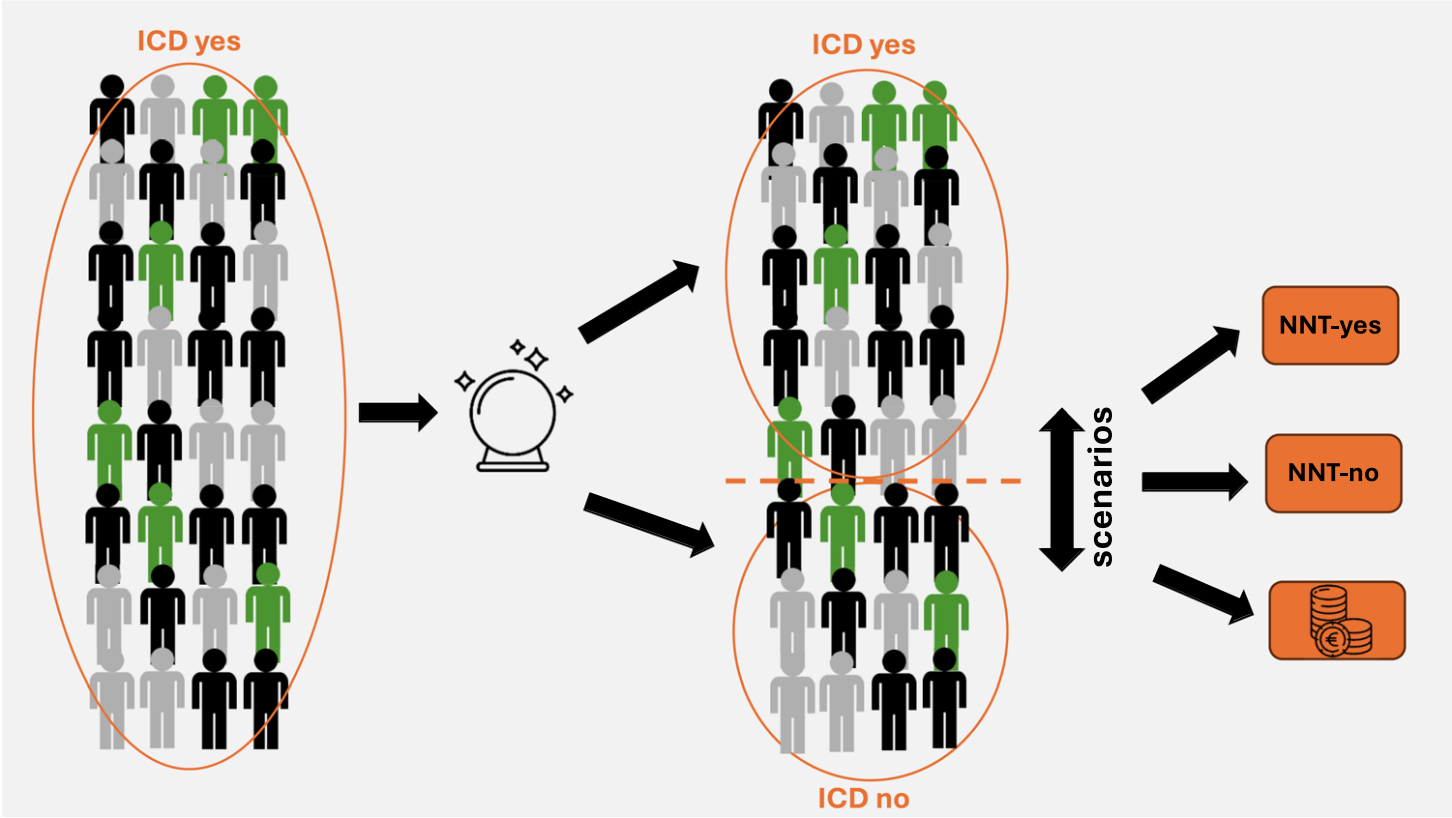

**Supplementary Information:**

The online version contains supplementary material available at 10.1007/s00392-025-02687-4.

## Introduction

Prophylactic implantable cardioverter defibrillator (ICD) implantation to prevent sudden cardiac death has repeatedly been shown not to benefit patients on the midterm while posing a risk of device-related complications and inappropriate shocks [1–4]. The cost-effectiveness of preventive treatment under current indication criteria remains uncertain and improved patient selection is deemed necessary from clinical and health economic perspectives [5].

The DO-IT was set up to prospectively register and monitor patients for at least 2 years after de novo primary prevention ICD implantation to identify the ones who actually benefitted during this period [6]. Two prediction models were developed, one for mortality and one for the occurrence of appropriate ICD therapy with death as a competing risk [7]. In patients likely to die early despite ICD implantation or because of co-morbid conditions, the implantation may be considered as non-beneficial or futile and may be foregone. This will reduce patient burden and save expenses of the procedure itself, the hospital admission(s) and treatment of possible complications [8, 9]. Likewise, patients alive and without further cardiac arrythmias likely do not receive appropriate ICD therapy in the first 2 years. Thus, without jeopardizing the patient’s health, immediate burden may be avoided, while expenses could at least be postponed safely. If a patients’ probability, i.e., eligibility for appropriate ICD therapy sufficiently increases at a later stage, ICD implantation could still be offered.

The performance of the prediction models was evaluated by assessing discrimination and calibration. Both internal and external validation showed good model performance for mortality. The predictive value of the appropriate ICD therapy model was lower than hoped for [7]. Importantly though, while discrimination and calibration are important aspects of model performance, the ability to make better decisions using models in view of their clinical and health economic implications for daily practice remains of utmost importance. [10, 11]

If predictive models for appropriate ICD therapy and mortality are to be used to guide treatment decisions, risk thresholds are required to classify patients as currently needing ICD implantation or not. For each alternative threshold in a stricter scenario the numbers needed to treat (NNT) to prevent one patient from dying can be assessed in the group of patients with an indication (NNT-yes; the lower the better) and in the group of patients without an indication (NNT-no; the higher the better). Likewise, the implied shifts in hospital health care expenditures can be assessed. We studied these implications by simulating stricter indication scenarios for primary prevention ICD implantation in the national DO-IT cohort of ICD recipients under existing indication criteria using the DO-IT models for the prediction of appropriate ICD therapy and mortality [7]. Scenarios were run for higher prior probabilities of receiving appropriate ICD therapy and lower risks of mortality (and their combinations) as risk thresholds, as well as for selected combinations of such thresholds.

These scenario analyses will aid stakeholders in health care to ration the demand for and supply of ICDs, thus contributing to sustainable (hospital) health care. [12]

## Methods

### The DO-IT registry and stricter ICD indication scenarios based on predicted risks

All 28 Dutch ICD-implanting centres contributed data to the DO-IT Registry on primary prevention ICD recipients with a reduced left ventricular ejection fraction. Patient and device characteristics were gathered at inclusion, while (in) appropriate ICD therapies [shock or anti-tachycardia pacing (ATP)], complications, interventions, hospital inpatient stay, disease courses and death were prospectively collected during at least 2 years of follow-up [6]. All registry data were extensively monitored.

All DO-IT patients received their ICD under the existing guidelines at the time (2014–2016) for primary prevention ICD-implantation, the reference situation. Associations between baseline characteristics and experiencing appropriate ICD therapy or mortality were studied to identify independent predictors [7]. Predicted risks of appropriate ICD therapy and of mortality were held against stricter risk thresholds scenarios to classify patients as “ICD indicated” or “ICD not (yet) indicated". Eight threshold scenarios to refrain from ICD implantation based on patients' risks of receiving appropriate ICD-therapy were: < 2%, < 2.5%, < 3%, < 4%, < 5%, < 7.5%, < 10%, < 15% per 2 years. Six threshold scenarios to refrain from ICD implantation based on patients' risks of dying during follow-up without benefitting from the ICD were: > 50%, > 40%, > 30%, > 25%, > 20%, > 15% per 2 years. Of the 48 possible combinations of both models the following five increasingly stricter combination of threshold scenarios to refrain from ICD implantation were assessed:

- Mortality risk > 50% or mortality risk ≤ 50% with < 2% appropriate ICD therapy risk
- Mortality risk > 40% or mortality risk ≤ 40% with < 2.5% appropriate ICD therapy risk
- Mortality risk > 30% or mortality risk ≤ 30% with < 3% appropriate ICD therapy risk
- Mortality risk > 25% or mortality risk ≤ 25% with < 4% appropriate ICD therapy risk
- Mortality risk > 20% or mortality risk ≤ 20% with < 5% appropriate ICD therapy risk

### Assessment tool, what-if scenarios and assumptions, numbers needed to treat

The 'DO-IT Budget impact 1.1' assessment tool was developed in Microsoft Excel to study the impact of the stricter ICD indication scenarios on NNT-yes, NNT-no, and hospital care expenditures.

In absence of a comparative study design for ethical reasons, 'what-if' scenarios of restricted access to ICD implantation were developed for distinct subgroups of patients in the DO-IT registry. Thirty-two subgroups were defined by combination of ICD device type [single chamber ICD, dual chamber ICD, cardiac resynchronizing therapy defibrillator (CRT-D), and subcutaneous ICD (S-ICD)], having received appropriate ICD therapy (yes/no), having experienced major ICD-related complication (or minor complication with admission) (yes/no), and dying (yes/no) during 2 years after ICD implantation. The observed distribution of patients across these subgroups in the DO-IT Registry was taken as the reference situation.

The what-if scenarios reflect what could reasonably have happened, if ICDs had not been implanted in the DO-IT patients. First, surviving patients without appropriate ICD therapy would—not having experienced significant ventricular arrhythmias—still have survived without an ICD. If these patients were to fail stricter ICD indication criteria, costs of the index intervention as well as possible treatment costs of complications and inappropriate ICD therapies would have been avoided.

Second, deceased patients without appropriate ICD therapy and without a major ICD-related complication would most likely also have died in absence of an ICD implantation. The same holds for patients without a major ICD-related complication who died despite appropriate ICD therapy. Hence, expenses for the implantation and for treatment of (in)appropriate ICD therapies in these subgroups would then have been avoided.

Third, deceased patients with a major ICD-related complication would have had a lower risk of dying in absence of ICD implantation. Indeed, five of 21 (or 23.8%) deceased patients with major ICD-related complication(s) in the whole cohort died because of that complication. We assumed these patients to survive in absence of ICD-implantation. The other patients whose deaths were not attributed to the major ICD-related complication(s), would also most likely have died in absence of ICD implantation.

Fourth, patients with a CRT indication, currently receiving a CRT-D, would in absence of an ICD indication have received a standard pacemaker (CRT-P) for treatment of their left ventricular asynchrony related heart failure. Hence, not implanting the defibrillator prevents the related expenses, but other expenditures then come in place.

Fifth, as with all prediction models, the DO-IT models generate 'false-negative predictions’: a low risk of appropriate ICD therapy is suggested for a surviving patient, while the patient actually experienced appropriate ICD therapy. Forecasting the counterfactual, i.e., what would have happened to these patients had they not been treated with an ICD under stricter indication criteria is complicated. We assumed that at least one out of three of these patients would die because of sudden cardiac death. This uncertain assumption was addressed in sensitivity analyses (see below).

The assumed 2-year mortality risks in absence of ICD implantation are shown for subgroups of patients in Table S2 of the supplementary material. The NNT-yes for the reference situation was calculated as the total number of patients in the DO-IT cohort divided by the ‘additional mortality’ as the product sum of differences in mortality risks per subgroup between ICD-implanted or not implanted situations of Table S2 and the distribution of patients over these subgroups. The NNT-yes and NNT-no for stricter indication scenarios were calculated likewise, but based on absolute proportions of indicated and not (yet) indicated patients in the total cohort and corresponding absolute proportional distributions over the subgroups of patients.

### Foregone and new expenses

Assumed shifts in use of hospital care and the related expenses have been stated explicitly. Table 1a shows the observed mean use of reimbursable hospital care resource packages—the 'diagnosis-treatment combinations' (DBC)—during the 2 years of follow-up of four subgroups of patients who received a single chamber ICD-implant. Table 1b shows the expected mean use of reimbursable hospital care packages in absence of ICD-implantation. The shifts in use of hospital care resources for all subgroups are reported in Tables S3a–S6b in the supplementary material. Mean volumes of regular follow-up care in absence of an indication for ICD-implantation were based on usual monitoring (watchful waiting), corrected—if applicable—for the observed mean time till death not caused by major ICD-related complication(s).

**Table 1:** Observed and assumed 2-year mean use of health care resources by four subgroups of single chamber ICD-implant patients in case of ICD implantation (A) and no ICD implantation (B)

DBC code	Description as applied in DO-IT registry	Device type	Single chamber			
		subgroup	1	2	3	4
		appropriate ICD-therapy	No	No	No	No
		major complication	No	No	Yes	Yes
A	ICD implantation	deceased	No	Yes	No	Yes
99,899,013	Pocket infection with a maximum of 5 inpatient days		0	0	0	0
99,899,027	Pocket infection with more than 28 inpatient days		0	0	0	0
99,899,028	Pocket infection with 6 to 28 inpatient days		0	0	0.167	0
99,899,030	Implantation of pacemaker with hospital admission		0	0	0	0
99,899,064	Admission with a maximum of 5 inpatient days for non-appropriate ICD-therapy		0.157	0.063	0.111	1
99,899,068	Admission with 6 to 28 inpatient days for non-appropriate ICD-therapy		0	0.063	0.111	0
99,899,070	Admission with a maximum of 5 inpatient days for appropriate ICD-therapy		0	0	0	0
99,899,079	Admission with more than 28 inpatient days for appropriate ICD-therapy		0	0	0	0
99,899,080	Admission with 6–28 inpatient days for appropriate ICD-therapy		0	0	0	0
109,599,007	Drainage of thorax with a maximum of 5 inpatient days		0	0	0.111	0
109,599,014	Drainage of thorax with 6 to 28 inpatient days		0	0	0	0
219,699,016	Regular follow-up care after ICD implantation per 120 days		6	3	6	5
219,699,027	Follow-up care after ICD implantation during first 90 days		1	1	1	1
979001242a	Implantation of dual chamber ICD including leads		0	0	0	0
979001242b	Implantation of CRT-D including leads (979001242a plus extra lead costs)		0	0	0	0
979,001,243	Implantation of single chamber AICD or S-ICD including leads		1	1	1.167	1
979001244a	Implantation or replacement of dual chamber ICD including leads		0	0	0	0
979001244b	Repositioning of lead or dual chamber ICD (979001244a minus device costs)		0	0	0	0
979001244c	Implantation or replacement of CRT-D including leads (979001244a plus extra lead costs)		0	0	0	0
979001244d	Repositioning of lead or CRT-D (979001244c minus device costs)		0	0	0	0
979001245a	Implantation or replacement of single chamber AICD including leads		0	0	0	0
979001245b	Repositioning of lead or single chamber AICD (979001245a minus device costs)		0	0	0.389	0
979001245c	Repositioning of lead or S-ICD (979001245a minus device costs)		0	0	0	0
979,001,246	Placement of new lead		0	0	0.056	0
979001258a	Extraction of lead or AICD (derived from 2016 reimbursement level)		0	0	0.167	0
979001258b	Replacement of lead with new lead (979001258a plus extra lead costs)		0	0	0.278	0
190,668*	LifeVest (add-on; *declaration code)		0	0	0.056	0
333000c*	Drainage pericard (add-on; *declaration code)		0	0	0	0

		Ventricular tachycardia	No	No	No	No
		deceased	No	Yes	No	Maybe
B	No ICD implantation	Assumed mortality risk	0	1	0	0.762
99,899,030	Implantation of pacemaker with hospital admission		0	0	0	0
99,899,045	Regular follow-up care during chronic heart failure per 120 days		7	4	7	6.238
219,699,014	Regular follow-up care after pacemaker implantation per 120 days		0	0	0	0
219,699,023	Follow-up care after pacemaker implantation during first 90 days		0	0	0	0

The tariffs for reimbursements were taken from www.opendisdata.nl of the Dutch Health Care Authority and represent trimmed mean charges over all hospital care providers during the latest calendar year with 100% national coverage. These mean trimmed charges were price-indexed for the year 2019 with yearly general consumer indices for the Netherlands (Statistics Netherlands, access month July 2019). If no trimmed mean charge was determined because of too infrequent use of the hospital care package (less than 100 observations nationally) available data from an earlier calendar year were used and also price-indexed for the base year 2019 (e.g., lead or automatic ICD extraction). Some adaptations were made to match a reimbursement level more closely with the actual use of hospital resources. For instance, add-on costs for an extra lead in case of CRT-D implantation and subtraction of the unit costs of the device itself was used, if the intervention was meant to just reposition a lead or an implantable device rather than adding new components. It was further assumed that the level of care during hospital admissions for (major) pocket infections best resembled the intensity of care for patients with endocarditis. Tariffs are reported in Table S7 in the supplementary material. The mean total reimbursements for patients during the follow-up period of 2 years or until death, whichever came first, are shown in Table 2 for, respectively, single chamber, dual chamber, CRT-D and subcutaneous device implants. The assumed reimbursements if a patient would not have had an ICD implanted and the total expenses per subgroup per ICD type are also reported. During follow-up we did not observe patients with a single chamber, CRT-D or S-ICD device implanted who received appropriate ICD therapy, experienced a major complication and died (subgroups 8, 16 and 32).

**Table 2:** Mean total reimbursement per subgroup for single chamber, dual chamber, CRT-D and S-ICD implant patients during observation (left) and assumed mean total reimbursement in absence of ICD-implantation (right)

	ICD implantation					No ICD implantation			
Device type	Subgroup	Appropriate ICD therapy	Major complication	Deceased	Mean reimbursement (€)	Major event	Deceased	Assumed mortality risk	Mean reimbursement (€)
Single chamber	1	No	No	No	23 348	No	No	0	3458
	2	No	No	Yes	22 507	No	Yes	1	1976
	3	No	Yes	No	37 641	No	No	0	3458
	4	No	Yes	Yes	24 294	No	Maybe	0.762	3082
	5	Yes	No	No	24 111	Yes	Maybe	0.333	2800
	6	Yes	No	Yes	26 095	Yes	Yes	1	1812
	7	Yes	Yes	No	33 362	Yes	Maybe	0.333	2471
	8	YES	Yes	Yes	0	Yes	Maybe	0.841	0^*^
Dual chamber	9	No	No	No	25 932	No	No	0	3458
	10	No	No	Yes	24 476	No	Yes	1	1482
	11	No	Yes	No	40 426	No	No	0	3458
	12	No	Yes	Yes	51 563	No	Maybe	0.762	2329
	13	Yes	No	No	26 748	Yes	Maybe	0.333	2964
	14	Yes	No	Yes	27 314	Yes	Yes	1	1482
	15	Yes	Yes	No	37 266	Yes	Maybe	0.333	2800
	16	Yes	Yes	Yes	25 204	Yes	Maybe	0.841	2462
CRT-D	17	No	No	No	26 946	No	No	0	15 371
	18	No	No	Yes	25 840	No	Yes	1	14 466
	19	No	Yes	No	42 776	No	No	0	15 371
	20	No	Yes	Yes	36 327	No	Maybe	0.762	14 682
	21	Yes	No	No	28 639	Yes	Maybe	0.333	14 969
	22	Yes	No	Yes	28 658	Yes	Yes	1	14 567
	23	Yes	Yes	No	32 522	Yes	Maybe	0.333	15 070
	24	Yes	Yes	Yes	0	Yes	Maybe	0.841	0^*^
S-ICD	25	No	No	No	23 104	No	No	0	3458
	26	No	No	Yes	22 012	No	Yes	1	1976
	27	No	Yes	No	31 704	No	No	0	3458
	28	No	Yes	Yes	26 707	No	Maybe	0.762	2329
	29	Yes	No	No	23 104	Yes	Maybe	0.333	2800
	30	Yes	No	Yes	22 740	Yes	Yes	1	2305
	31	Yes	Yes	No	26 244	Yes	Maybe	0.333	3129
	32	Yes	Yes	Yes	0	Yes	Maybe	0.841	0^*^

*During follow-up we did not observe patients who received appropriate ICD therapy and experienced a major complication and died with a single chamber, CRT-D or S-ICD implanted (subgroup 8, 16 and 32)

### Expected yearly incidence of presenting cases

The number of patients with a reduced left ventricular function in a setting of structural heart disease in the Netherlands was not immediately available from existing registries. Reports over the years from the Netherlands Heart Foundation, Netherlands Heart Registry, and National Cardiac Data Registry (NCDR) were imperfect due to different levels of completeness of data provision by local hospital health care providers. The gradual inclusion of hospitals in the DO-IT registry over the calendar years too did not result in a ‘gold standard’ for determining the absolute number of presenting cases at national scale. Instead, the national health care database for reimbursement data (www.opendisdata.nl), managed by the Dutch Health Care Authority, and the NCDR 2015 report were used to identify distinct patient numbers [13]. This resulted in a yearly incidence of 2500 cases for primary prevention ICD implantation in the Netherlands. For more details on this count, see supplementary material Table S8 and its subsequent text.

### Health economic benefits of stricter indications

The budget impact for each stricter scenario to refrain from ICD-implantation was determined by multiplying the yearly number of presenting cases with the product sum of (i) the absolute proportions of patients of the total study cohort without ICD indication for the 29 observed subgroups of patients in Table 2 and (ii) the subgroup-specific differences between mean reimbursements given ICD implantation and no ICD implantation.

The analysis was incidence-based, meaning that reimbursements during 2 years of follow-up were assigned to the year of first presentation. Because the high reimbursements for ICD implantation are claimed during the year of presentation and because of the projected flat yearly number of presenting cases, the incidence-based approach will largely coincide with a prevalence-based approach to estimate the budget impact.

The budget impact of each stricter scenario was reflected in mean savings in reimbursement in € per presenting case compared to the existing guidelines, total yearly national savings in €, and the latter expressed as a percentage of the spendings in the reference or base case scenario under the existing guidelines.

To further stimulate the debate on the more societal value of using the (combination of) prediction models in daily practice for indication purposes, the potential savings in reimbursements per additional life lost and per lost quality adjusted life year (QALY) are calculated.

### Sensitivity analysis for NNTs

As the most realistic lethal consequences of not implanting an ICD in subpopulations according to the stricter indication criteria scenarios it was assumed that 23.8% of patients who died with a major ICD-related complication would survive and that one-third of patients alive with appropriate ICD therapy would die. These assumptions were addressed in a sensitivity analysis for the NNTs by considering two scenarios in favor of ICD implantation, an optimistic and unrealistically optimistic one. In the optimistic scenario it was assumed that fewer deaths among patients with a major ICD-related complication were attributable to that major complication: 10.6% as the lower 95% confidence limit of the 23.8%. In addition, 50% of patients alive with an ICD implanted and having experienced appropriate ICD therapy would have died in absence of the ICD. In the unrealistically optimistic scenario, it was assumed that a major ICD-related complication never caused death in deceased patients having experienced such complication and that all patients having experienced appropriate ICD-therapy would have died in absence of the ICD.

## Results

### Patients and predicted risks of appropriate ICD therapy and mortality

Between September 2014 and May 2016, 1443 patients were included in the DO-IT registry. The mean age at inclusion was 65.9 (SD 10.2), 72% was male, 14% presented with NYHA functional class I, 63% NYHA-II and 23% NYHA-III/IV. Single chamber ICD devices were implanted in 33%, dual chamber in 16%, CRT-D in 43% and subcutaneous in 8%. For further details on baseline characteristics, see Table S1 of the supplementary material. Two patients were lost to follow-up, but still alive in May 2019. One of them did have a complication which resulted in the ICD being explanted. For the reference situation both patients were considered as not having experienced an appropriate therapy (prior probabilities of not having so of 0.84 and 0.92 as predicted by our appropriate therapy model). Within 2 years of follow-up 132 (9.15%) patients experienced appropriate ICD-therapy, 114 (7.90%) experienced at least one major complication and 131 (9.08%) patients died.

The predicted model-based individual risks for the DO-IT ICD recipients of (i) receiving appropriate ICD therapy or (ii) dying within 2 years of follow-up ranged from 0.89% to 66.67%, respectively, from 0.16% to 75.51%. The distributions of these predicted risks in the registry cohort are shown in Figure S1a, b in the supplementary material.

### Clinical impact

Table 3 shows the realistic clinical impact of stricter indication scenarios to refrain from ICD implantation. The proportions of patients with too low individual risks of appropriate ICD therapy ranged from 0.062 to 0.858, respectively, whereas proportions of patients with too high individual mortality risks ranged from 0.009 to 0.176. For the combined scenarios the proportions ranged from 0.071 to 0.339. Expected excess mortalities ranged from 0.02% to 1.54% under the appropriate ICD therapy risk scenarios, from 0.02% to 0.29% under mortality risk scenarios, and from 0.04% to 0.27% under the selected combined scenarios.

**Table 3:** Clinical impacts of stricter indication scenarios to refrain from primary prevention ICD-implantation based on minimum appropriate ICD-therapy risks, maximum mortality risk, and selected combinations

Scenarios	Proportion of patients without indication for ICD implantation	Mortality (as %)	NNT-no^1^	NNT-yes^2^
Observed base case	0	9.08	–	42
		Expected mortality risk (as %)		
Appropriate ICD-therapy risk				
< 2%	0.062	9.10	271	40
< 2.5%	0.116	9.13	220	38
< 3%	0.168	9.18	170	37
< 4%	0.214	9.18	217	35
< 5%	0.260	9.18	246	33
< 7.5%	0.452	9.57	93	29
< 10%	0.665	10.01	72	23
< 15%	0.858	10.62	56	17
Mortality risk				
> 50%	0.009	9.10	40	42
> 40%	0.019	9.10	85	42
> 30%	0.040	9.15	59	42
> 25%	0.067	9.17	73	41
> 20%	0.113	9.25	68	40
> 15%	0.176	9.37	61	40
Combined scenarios				
> 50% mortality risk or ≤ 50% mortality risk with < 2% appropriate ICD-therapy risk	0.071	9.12	154	40
> 40% mortality risk or ≤ 40% mortality risk with < 2.5% appropriate ICD-therapy risk	0.135	9.15	178	38
> 30% mortality risk or ≤ 30% mortality risk with < 3% appropriate ICD-therapy risk	0.203	9.25	121	36
> 25% mortality risk or ≤ 25% mortality risk with < 4% appropriate ICD-therapy risk	0.267	9.27	140	34
> 20% mortality risk or ≤ 20% mortality risk with < 5% appropriate ICD-therapy risk	0.339	9.35	124	32

^1^Number needed to treat in subpopulation of patients without indication for ICD implantation

^2^Number needed to treat in subpopulation of patients still indicated for ICD implantation

In the base case scenario in which all patients have an ICD implanted, we observed an NNT of 42, meaning that a sudden cardiac death was prevented during 2 years of follow-up in one of every 42 patients with an ICD implanted. In the appropriate ICD therapy risk, mortality risk and combined scenarios in the patients still indicated with an ICD, the NNT-yes ranged from 17 to 40, from 40 to 42, and from 32 to 40, respectively. In contrast, the NNT-no ranged from 56 to 271, from 40 to 85, and from 124 to 178 under the appropriate ICD therapy risk, mortality risk and selected combined scenarios, respectively. For instance, if all patients with a risk of appropriate therapy over 2 years below 5% would not receive an ICD implanted, then one in every 246 would experience sudden death. Yearly, 0.26*2500 or 650 ICD implantations for primary prevention would be foregone, but 2.64 extra patients on average would die.

**Fig. 1:**
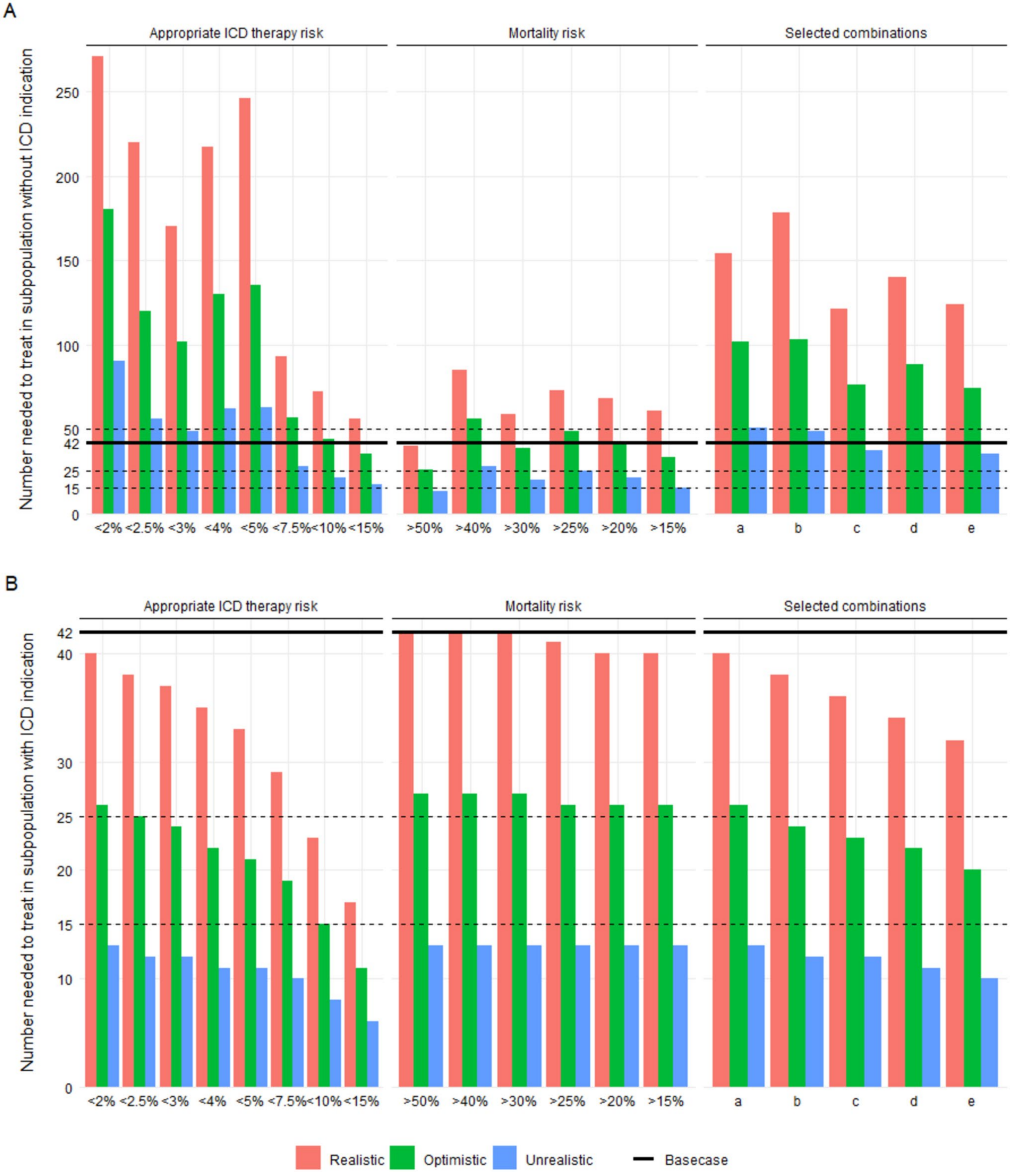
Numbers needed to treat to prevent sudden cardiac death, given realistic, optimistic, and unrealistic lethal consequences of no ICD implantation, for subpopulations of patients without (NNT-no, **A**) or with (NNT-yes, **B**) an indication for ICD implantation under stricter indication scenarios to refrain from ICD implantation

Figure 1 shows the results of the sensitivity analysis. From the optimistic perspective the NNT-yes ranged from 11 to 26, from 26 to 27, and from 20 to 26 under the appropriate ICD therapy risk, mortality risk and selected combined scenarios, respectively; from the unrealistic perspective the NNT-yes ranged from 6 to 13, from 13 to 13, and from 10 to 13, respectively. From the optimistic perspective the NNT-no ranged from 35 to 180, from 26 to 56, and from 73 to 103 under the appropriate ICD therapy risk, mortality risk and selected combined scenarios, respectively; from the unrealistic perspective the NNT-no ranged from 17 to 90, 13 to 28, and from 35 to 51, respectively.

### Economic impact

Table 4 shows the economic and budget impact of stricter indication scenarios. In the base case scenario, the expected yearly incidence-based reimbursement per presenting case was €26,379 or nearly €66 million per 2,500 patients.

**Table 4:** Economic and budget impacts of stricter indication scenarios to refrain from primary prevention ICD-implantation based on minimum appropriate ICD-therapy risks, maximum mortality risk, and selected combinations

Scenarios	Reimbursement (€) per presenting case	Total yearly reimbursement (€)		
Observed base case	26 379	65 946 427	0	0
	Mean savings (€) per presenting case	Total yearly savings (€), incidence-based	Yearly savings %	Mean savings (€) per extra deceased case
Appropriate ICD-therapy risk				
< 2%	989	2 471 893	3.8	4 278 676
< 2.5%	1847	4 617 396	7.0	3 497 329
< 3%	2825	7 062 904	10.7	2 852 878
< 4%	3645	9 113 394	13.8	3 681 122
< 5%	4411	11 027 713	16.7	4 176 330
< 7.5%	7652	19 129 524	29.0	1 566 230
< 10%	11 549	28 873 545	43.8	1 240 697
< 15%	15 158	37 895 900	57.5	985 393
Mortality risk				
> 50%	136	339 016	0.5	586 814
> 40%	304	761 201	1.2	1 317 586
> 30%	624	1 558 998	2.4	899 506
> 25%	1024	2 560 025	3.9	1 107 806
> 20%	1803	4 506 541	6.8	1 070 721
> 15%	2932	7 329 866	11.1	998 095
Combined scenarios				
> 50% mortality risk or ≤ 50% mortality risk and < 2% appropriate ICD-therapy risk	1116	2 790 828	4.2	2 415 366
> 40% mortality risk or ≤ 40% mortality risk and < 2.5% appropriate ICD-therapy risk	2143	5 358 517	8.1	2 823 262
> 30% mortality risk or ≤ 30% mortality risk and < 3% appropriate ICD-therapy risk	3386	8 466 147	12.8	2 011 495
> 25% mortality risk or ≤ 25% mortality risk and < 4% appropriate ICD-therapy risk	4466	11 165 102	16.9	2 332 571
> 20% mortality risk or ≤ 20% mortality risk and < 5% appropriate ICD-therapy risk	5688	14 219 384	21.6	2 076 001

Under the appropriate ICD therapy risk scenarios, savings per presenting case ranged from €989 to €15,158 with associated total yearly incidence-based savings per calendar year ranging from €2.47 million to €37.9 million, comprising between 3.8% and 57.5% of the yearly expenditures. Savings per presenting case under the mortality risk scenarios ranged from €136 to €2,931 with associated total yearly incidence-based savings per calendar year ranging from €0.34 million to €7.3 million, or between 0.5% and 11.1% of the yearly expenditures. Under the selected combined risks scenarios, savings per presenting case range from €1,116 to €5,687 with associated total yearly incidence-based savings per calendar year ranging from €2.8 million to €14.2 million, or between 4.2% and 21.6% of the yearly expenses.

### Savings per additional life lost

The savings per additional life lost (Table 4) as the trade-off between less ICD implantations and increased mortality range from €1.0 to €4.3 million, from €0.6 to €1.4 million, and from €2.0 to €2.8 million under the stricter appropriate ICD therapy, mortality and selected combined risks scenarios. For instance, if patients below a 5% risk over 2 years of receiving appropriate ICD therapy would be ineligible for ICD implantation (with the NNT-no of 246), then the yearly hospital care budget for primary prevention of sudden cardiac death would be reduced by €11 million (16.7%) or almost €4.2 million per life lost.

## Discussion

Improvement in patient selection criteria for primary prevention ICD implantation is deemed necessary. Using the developed and internally validated DO-IT prediction models of appropriate ICD-therapy and/or mortality to finetune current primary prevention ICD implantation guidelines in the Netherlands is opportune. Even if prediction models showed a restricted discriminative performance at individual level, the clinical value at a cohort level became evident. Subpopulations with high numbers needed to treat to prevent sudden cardiac death could be identified, in which ICD implantation should perhaps be foregone or delayed (NNT-no). Withholding ICD implantation in these patients improves the efficiency of primary prevention of sudden cardiac death by lowering the NNT among the patients who remain eligible for ICD implantation (NNT-yes) and saving hospital care expenditures of ICD implantation, in-patient hospital stay and treatment of ICD-related complications.

There are several thresholds for treatment indication suggested by either model and by the combination of models that reduce hospital health care expenditures considerably. The trade-off by excess mortality remains below or near 1 in 1000 patients for 10 of the 19 studied scenarios. Adjusting guidelines by increasing the minimum probability of receiving appropriate ICD therapy within 2-year post-implant is most promising financially, and those scenarios are perhaps also the most acceptable ones from a clinical perspective, i.e., to base implantation decisions on the likelihood of receiving appropriate ICD therapy. Furthermore, the results show that many patients have to undergo ICD implantation without apparent immediate benefit to save one person from sudden cardiac death. For all thresholds up to 5% for the minimal probability of appropriate *therapy* the NNT-no was ≥ 170 under the realistic and ≥ 101 under the optimistic scenarios. Even under the unrealistically optimistic scenario in which ICD implantation would never be associated with a life-ending complication and in which all patients who received ICD therapy would die in absence of an ICD, the NNT-no would still be at least 49. To substantiate that the latter scenario is unrealistic, one should note that primary prevention trials consistently demonstrated that the number of appropriate *shocks* exceeds the sudden death and overall mortality rate in the control group, making appropriate ICD shock as a perfect surrogate for sudden cardiac death unlikely [14–17]. In the DEFINITE trial twice as many appropriate shocks were reported in the ICD arm compared to fatal events in the control group [15]. Experts of the DO-IT steering committee advised to assume a case–fatality rate of 1 out of 3 (0.333) in absence of ICD implantation as most realistic, because appropriate ICD therapy as subgroup classifier in the DO-IT registry included ICD shocks as well as anti-tachycardia pacing. [18] If stricter ICD selection is applied, it is to be expected that SCD occurs in small number of patients who will not receive an ICD. This is inherent in the search for better selection criteria. To illustrate, currently there are also cardiac patients (e.g., with higher LVEF), where we accept this risk, and they too are not within our current, broad selection for primary prevention of SCD.

Whether or not the NNT-no presented at each scenario is 'acceptable' remains subjective. Jolly and colleagues suggested that an NNT of 50 is of clinical significance and that an *annual* baseline risk of arrhythmic death of 3% is required for a patient to benefit from device implantation [19]. If this reasoning holds, then a cutoff of a minimum 5% *biannual* probability of appropriate therapy with an NNT-no of 246, a 16.7% budget cut and €4.18 million saved per extra deceased case seems defensible. In addition, nearly 8% of those 246, or 19 patients would not experience otherwise inflicted harm from major ICD-related complications [8].

To further put the €4.18 million saved per extra deceased case of this scenario into perspective and facilitate health policy decision making, one might consider the following. In the Netherlands regulatory authorities agreed upon a maximum threshold for reimbursement of health technologies of €80,000 per quality adjusted life year (QALY or 1 year in perfect health) [20]. This threshold holds for patient populations with a high disease burden if left untreated. Although such high disease burden may not necessarily reflect the health status of patients indicated for primary prevention ICD implantation, one could conservatively state that at least more than this upper limit should be saved per QALY lost to make a more restrictive policy of access to ICD implantation acceptable from a societal perspective [21, 22]. Given the mean ICD implantation age of 65 years of the DO-IT target population with 72% being male, one may derive with the Disease Burden Calculator that these ICD recipients on average generate less than 20 QALYs during the remainder of life (according to the calculator: 16.4 QALYs without having a disease) [23]. If 20 QALYs at maximum would be lost per extra deceased case with €4.18 million being saved, then €209,000 would be saved per QALY lost. This amount is of societal significance and demonstrates the need for updated treatment guidelines for ICD implantation. This is also endorsed by the Dutch National Health Care Institute which recently restricted ICD indication for non-ischemic patients; recommending ICDs only in patients with pathogenic mutations or cardiac fibrosis. Likewise, several studies are currently ongoing in which primary prevention patients who fulfil the international guidelines are randomized to treatment with or without an ICD to assess both clinical and cost-effectiveness of ICD therapy to eventually adjust the current guidelines [24–27]. The results of this study may contribute to these initiatives.

Several limitations need to be considered. First, a budget impact analysis is challenging in a non-randomized study in which all patients actually received an ICD for primary prevention, because the counterfactual remains unobserved, i.e., it is not possible to tell with absolute certainty what care would have been provided in absence of the ICD implantation. This also requires that sufficient information can be obtained about the clinical discipline to which the care provision and costs can be attributed and about the extent in which comorbidities play a role in the care provided by the cardiologist. Staying on the conservative side we only included the costs that were certainly directly related to the ICD: ICD implantation, follow-up and treatment for ICD-related complications and ICD therapy. However, we did not include the costs of the unobserved pacemaker-related complications in patients with a CRT indication. We only incorporated the costs of pacemaker implantation and expected costs of monitoring by the cardiologist. Although literature shows that fewer complications occur after pacemaker implantation, the exception should be viewed as a limitation [28, 29]. Also, we decided by study protocol to ignore the use of non-hospital resources and related costs, because treatment and monitoring of ICD implanted patients is mainly provided by cardiologists and electrophysiologists. Hence, the costs and the potential costs savings presented in this paper reflect the hospital perspective and are an underestimation of the actual health care costs, but probably to a modest extent.

In addition, it is assumed that patients who would not meet the indication criteria and forego the ICD implantation generate reductions in reimbursement. These patients have chronic heart failure with a possible need for ICD-implantation at a later date in their life. It is yet uncertain if and when that day would come. An additional scenario of planned implantation on the longer term (after 2 years) after initial postponement seems unfeasible; most professionals would vote for actual health status driven decisions for ICD-implantation. However, the exclusion of costs related to out-of-hospital cardiac arrest, patient recovery and potentially secondary prevention ICD implantation is a limitation. These are unobserved and, therefore, beyond the scope of this paper but should be considered in further—preferably comparative—research and rationing decisions.

Furthermore, the prediction models target unnecessary ICD-implantations by identifying patients with a sufficiently low probability of experiencing a ventricular arrhythmia requiring ICD intervention. We did not account for the limited absolute number of misclassified patients who would not have an ICD implanted according to the stricter indication scenarios, while they received appropriate ICD therapy. After the hypothetical ‘no-implant’ decision they would experience a ventricular arrythmia with a 2 out of 3 chance to survive this event and, if so, would receive an ICD-implantation for secondary prevention.

In addition, the BIA was based on individual follow-up of patients for 2 years. Arrhythmic events will, however, also occur after 2 years of follow-up, and longer follow-up duration will result in a lower NNT [30]. Notably, with the developed prediction models we do not aim to give a definite advice to implant or not to implant an ICD based on their baseline risk, but merely to postpone ICD treatment for 1 or 2 years. The patients will still be under clinical follow-up and their risk will be evaluated yearly. It may be that ICD placement can be safely postponed for 2 years, and if a patients’ situation changes for the worse an ICD may still be implanted at a later stage. However, some patients will eventually not receive an ICD, since patients are still at risk for death from causes other than arrhythmia.

Furthermore, we performed a budget impact analysis in the Dutch primary prevention patient population with a given distribution of patients across subgroups by experiencing appropriate ICD therapy, experiencing a complication, death and implanted device type. Several factors, such as implanted device types used, (definitions of) occurrences of (in)appropriate ICD therapy and complications, and reimbursement tariffs may differ in other cohorts or healthcare systems, which would affect the calculated cost savings and NNTs. Our findings should be interpreted within the high-cost Dutch healthcare setting and may not be directly generalizable to countries with different healthcare expenditure levels. On request of the funding agencies, we additionally have calculated the potential cost savings based on a (bottom up) micro-costing approach in which the resources used as observed in the study are valued using unit costs to obtain total costs per patient [31]. This cost minimization analysis leads to approximately the same conclusions, but with on average 9–14% lower cost savings per presenting case in each scenario than was reported in this paper.

Finally, it should be noted that improvements in heart failure treatment, since the setup of the DO-IT study (e.g., the introduction of SGLT2 inhibitors and sacubitril/valsartan) has not been accounted for in our analyses. These therapies are now more widely adopted and have been shown to significantly reduce the risk of heart failure-related events, including potentially fatal arrhythmias. As a result, the likelihood of appropriate ICD therapy is expected to be even lower in today's patient population compared to the DO-IT cohort. This could affect both the estimated clinical benefits of ICD therapy and the size and timing of future budget impact assessments, depending on national diffusion rates. Therefore, while the absolute event rates in this study may be somewhat higher than would be observed in a contemporary cohort, the implications of our findings—namely, the importance of improved risk stratification and the potential for cost savings—are likely even more relevant in the current clinical landscape.

In conclusion, from a socioeconomic perspective improvement of the primary prevention ICD implantation guidelines in the Netherlands is warranted. The results show that cost savings would be substantial even under the five least strict scenarios for the probability of receiving appropriate ICD therapy. We believe that the current ‘one size fits all’ approach to defibrillator implantation does not represent effective nor efficient use of scarce health care resources. The trade-off between risk and benefit remains suboptimal. We believe that the prediction models can be of added value in the clinical decision to implant an ICD. In a situation of significant uncertainty regarding benefits, referring to a model, even though with modest predictive value at individual level, is in our opinion the optimal approach to improve selection of patients, because at the cohort level there is ample room for improvement from a clinical as well as economic perspective. There is room for more efficient ICD indication criteria, better outcomes and better use of limited health care resources. If ICD implantations are better allocated than under the current guidelines, this will improve the clinical effectiveness (decrease of NNT-yes) and consequently the cost-effectiveness of ICD therapy. The presented results are clinically intuitive and help relate derived benefit of primary prevention ICD implantation back to rational decision making in daily clinical practice. It may offer guidance to select an optimal risk threshold and help facilitate communication and discussion among cardiologists, patients, patient representatives, insurers, health care authorities and policy makers to better understand the clinical and health economic implications of implanting ICDs under the current guidelines to the total eligible population.

## Supplementary Information

Below is the link to the electronic supplementary material.

Supplementary file1 (DOCX 110 KB)

## Data Availability

Data underlying this article will be shared on reasonable request to the corresponding author.
