# Supplementary material for: Changes in numbers needed to treat and hospital care expenditures of optimized indications for primary prevention implantable cardioverter defibrillators: a scenario analysis
Source: Clin Res Cardiol. 2025 Jun 10;115(4):576–89. doi: 10.1007/s00392-025-02687-4 (PMC13013116; doi:10.1007/s00392-025-02687-4)
Supplement: Supplementary file 1 — Supplementary file1 (DOCX 110 KB) [file 392_2025_2687_MOESM1_ESM.docx]

**Electronic Supplementary material**

This supplementary material has been provided by the authors to give readers additional information about their work.

Supplement to: M. van Barreveld^1,2^, P.F.H.M. van Dessel^3^, E. Buskens^4^, L.V.A. Boersma^5^, P.P.H.M. Delnoy^6^, A.E. Tuinenburg^7^, D.A.M.J. Theuns^8^, P.H. van der Voort^9^, G.P. Kimman^10^, T.E. Verstraelen^1^, A.H. Zwinderman^2,11^, A.A.M. Wilde^1^, M.G.W. Dijkgraaf^2,11^. Changes in number needed to treat and hospital care expenditures of optimized indications for primary prevention implantable cardioverter defibrillators: a scenario analysis.

^1^Amsterdam UMC location University of Amsterdam, Cardiology, Meibergdreef 9, Amsterdam, Netherlands, ^2^Amsterdam UMC location University of Amsterdam, Epidemiology and Data Science, Meibergdreef 9, Amsterdam, Netherlands, ^3^Medisch Spectrum Twente, Enschede, the Netherlands, ^4^Department of Epidemiology, University Medical Centre Groningen, Groningen, the Netherlands, ^5^Cardiology department, St. Antonius Ziekenhuis Nieuwegein, the Netherlands, ^6^Department of Cardiology, Isala Klinieken, Zwolle, the Netherlands, ^7^Department of Cardiology, Division of Heart and Lungs, University Medical Centre, Utrecht, the Netherlands, ^8^Department of Cardiology, Erasmus MC, Rotterdam, the Netherlands, ^9^Department of Cardiology, Catharina Ziekenhuis Eindhoven, Eindhoven, the Netherlands, ^10^Department of Cardiology, Noordwest Ziekenhuisgroep, Alkmaar, the Netherlands, ^11^Methodology, Amsterdam Public Health, Amsterdam, the Netherlands

Corresponding author:

M. van Barreveld

ORCHID iD: 0000-0002-4672-2477

m.vanbarreveld@amsterdamumc.nl, 0031 20 5669111

Meibergdreef 9, 1105 AZ, Amsterdam, J1B-207

Last update: April 14, 2025

**Table S1.** Baseline characteristics

| **Baseline variables**^*^ | **DO-IT (n=1443)** |
| --- | --- |
| Sex (men) | 1044 (72) |
| Age | 65.9 (10.2) |
| Academic implanting hospital | 487 (34) |
| BMI (*n*=1422) | 27.3 (4.7) |
| NYHA functional class (*n*=1435) |  |
| I | 207 (14) |
| II | 905 (63) |
| III/IV | 323 (23) |
| Ischaemic | 882 (61) |
| LVEF (*n*=1436) | 26.1 (6.2) |
| RV function (normal) (*n*=1221) | 867 (71) |
| HF hospitalisation <1 year (*n*=1430) | 303 (21) |
| Prior cardiac surgery | 371 (26) |
| NS-VT (*n*=1395) | 170 (12) |
| MR Severity (*n*=1336) |  |
| None | 880 (66) |
| Moderate | 290 (22) |
| Severe | 166 (12) |
| CVA/TIA (*n*=1414) | 185 (13) |
| Vascular disease (*n*=1321) | 291 (22) |
| Atrial fibrillation (*n*=1415) | 438 (31) |
| COPD (*n*=1422) | 211 (15) |
| Hypertension (*n*=1409) | 618 (44) |
| Diabetes Mellitus (*n*=1434) | 386 (27) |
| Hypercholesterolemia (*n*=1372) | 594 (43) |
| Familial SCD (*n*=1152) | 176 (12) |
| Smoking^†^ (*n*=1237) | 846 (68) |
| Betablocker | 1232 (85) |
| Diuretic | 1032 (72) |
| Aldosterone antagonist | 667 (46) |
| ACEi or ARB | 1288 (89) |
| Oral anticoagulant | 684 (47) |
| Digoxin | 143 (10) |
| Statin | 976 (68) |
| Heart rate (*n*=1424) | 71.5 (14.5) |
| PR Interval (*n*=1413) |  |
| Normal | 832 (59) |
| PR prolongation | 304 (21) |
| Not applicable | 277 (20) |
| QRS axis (*n*=1332) |  |
| Normal | 849 (64) |
| Left | 407 (31) |
| Right | 56 (4) |
| Extreme | 20 (1) |
| QRS duration (*n*=1359) |  |
| <120 ms | 614 (45) |
| 120-150 ms | 333 (25) |
| >150 ms | 412 (30) |
| QTc bazett (*n*=1421) | 465.1 (47.9) |
| QRS morphology (*n*=1355) |  |
| Normal | 582 (43) |
| LBBB | 496 (36) |
| RBBB | 77 (6) |
| Aspecific | 200 (15) |
| eGFR (*n*=1405) | 61.3 (18.9) |
| Sodium (*n*=1402) | 139.4 (3.0) |
| Potassium (*n*=1401) | 4.4 (0.4) |
| Hemoglobin (*n*=1357) | 8.6 (1.0) |
| NT-pro-BNP (*n*=588) | 338.9 (570.0) |
| Previous Pacemaker | 76 (5) |
| Device type |  |
| Single chamber | 480 (33) |
| Dual chamber | 231 (16) |
| CRT-D | 623 (43) |
| S-ICD | 109 (8) |
| Vascular access (*n*=1438) |  |
| Subclavia | 707 (49) |
| Other | 622 (43) |
| None | 109 (8) |

*BMI* body mass index; *NYHA* New York Heart Association; *LVEF* left ventricular ejection fraction; *RV* right ventricular; *HF* heart failure; *NS-VT* non-sustained ventricular tachycardia; *MR* mitral regurgitation; *CVA* cerebrovascular accident; *TIA* transient ischaemic attack; *COPD* chronic obstructive pulmonary disease; *SCD* sudden cardiac death; *ACEi* angiotensin converting enzyme inhibitor; ARB angiotensin receptor blockers; *QTc* heart rate-corrected QT; *LBBB* left bundle-branch block; *RBBB* right bundle-branch block; *eGFR* estimated glomerular filtration rate; *NT-pro-BNP* N-terminal pro B-type natriuretic peptide; *CRT-D* cardiac resynchronization therapy defibrillator; *S-ICD* subcutaneous implantable cardioverter defibrillator.

^*^Data are presented as mean (standard deviation) or number (percentage).

^†^Smoking, current or former smoker.

**Table S2**. Assumed 2-year mortality risks for subgroups of ICD-implant patients and if no ICD would be implanted

| **ICD implantation** |  |  |  |  |  |  |  |  |
| --- | --- | --- | --- | --- | --- | --- | --- | --- |
| appropriate ICD therapy | no | no | no | no | yes | yes | yes | yes |
| major complication | no | no | yes | yes | no | no | yes | yes |
| deceased | no | yes | no | yes | no | yes | no | yes |
|  |  |  |  |  |  |  |  |  |
| **No ICD implantation** |  |  |  |  |  |  |  |  |
| ventricular tachycardia | no | no | no | no | yes | yes | yes | yes |
| mortality risk | 0 | 1 | 0 | 0.762 | 0.333 | 1 | 0.333 | 0.841 |

Table S1 shows the assumed 2-year mortality risk for subgroups of ICD-implantation patients, if no ICD were to be implanted. We assumed the implantation of a CRT-P, if, according to the prediction model(s), the ICD is not indicated for a patient who would otherwise have had an CRT-D implanted under the current guidelines.

**Table S3**. Observed and assumed 2-year mean use of health care resources by subgroups of single chamber ICD-implant patients in case of ICD implantation (A) and no ICD implantation (B) **Table S4**. Observed and assumed 2-year mean use of health care resources by subgroups of dual chamber ICD-implant patients in case of ICD implantation (A) and no ICD implantation (B)

**Table S5**. Observed and assumed 2-year mean use of health care resources by subgroups of CRT-D implant patients in case of ICD implantation (A) and no ICD implantation (B) **Table S6**. Observed and assumed 2-year mean use of health care resources by subgroups of S-ICD implant patients in case of ICD implantation (A) and no ICD implantation (B)

| **DBC code** | **Description as applied in DO-IT Registry** | **Tariff 2019 (€)** |
| --- | --- | --- |
| **ICD implantation** | |  |
| 99899013 | Pocket infection with a maximum of 5 inpatient days | 2111 |
| 99899027 | Pocket infection with more than 28 inpatient days | 21154 |
| 99899028 | Pocket infection with 6 to 28 inpatient days | 7400 |
| 99899030 | Implantation of pacemaker with hospital admission | 13385 |
| 99899064 | Admission with a maximum of 5 inpatient days for non-appropriate ICD-therapy | 1555 |
| 99899068 | Admission with 6 to 28 inpatient days for non-appropriate ICD-therapy | 6365 |
| 99899070 | Admission with a maximum of 5 inpatient days for appropriate ICD-therapy | 1903 |
| 99899079 | Admission with more than 28 inpatient days for appropriate ICD-therapy | 21559 |
| 99899080 | Admission with 6 to 28 inpatient days for appropriate ICD-therapy | 7545 |
| 109599007 | Drainage of thorax with a maximum of 5 inpatient days | 2891 |
| 109599014 | Drainage of thorax with 6 to 28 inpatient days | 7301 |
| 219699016 | Regular follow-up care after ICD implantation per 120 days | 364 |
| 219699027 | Follow-up care after ICD implantation during first 90 days | 182 |
| 979001242a | Implantation of dual chamber ICD including leads | 23566 |
| 979001242b | Implantation of CRT-D including leads (979001242a plus extra lead costs) | 24566 |
| 979001243 | Implantation of single chamber AICD or S-ICD including leads | 20738 |
| 979001244a | Implantation or replacement of dual chamber ICD including leads | 19146 |
| 979001244b | Repositioning of lead or dual chamber ICD (979001244a minus device costs) | 10811 |
| 979001244c | Implantation or replacement of CRT-D including leads (979001244a plus extra lead costs) | 20146 |
| 979001244d | Repositioning of lead or CRT-D (979001244c minus device costs) | 11874 |
| 979001245a | Implantation or replacement of single chamber AICD including leads | 18034 |
| 979001245b | Repositioning of lead or single chamber AICD (979001245a minus device costs) | 10259 |
| 979001245c | Repositioning of lead or S-ICD (979001245a minus device costs) | 3141 |
| 979001246 | Placement of new lead | 5060 |
| 979001258a | Extraction of lead or AICD (derived from 2016 reimbursement level) | 8953 |
| 979001258b | Replacement of lead with new lead (979001258a plus extra lead costs) | 9953 |
| 190668* | LifeVest (add-on; *declaration code) | 2000 |
| 333000c* | Drainage pericard (add-on; *declaration code) | 1756 |
|  |  |  |
| **No ICD implantation** | |  |
| 99899030 | Implantation of pacemaker with hospital admission | 13385 |
| 99899045 | Regular follow-up care during chronic heart failure per 120 days | 494 |
| 219699014 | Regular follow-up care after pacemaker implantation per 120 days | 302 |
| 219699023 | Follow-up care after pacemaker implantation during first 90 days | 177 |

**Table S7**. Reimbursement levels of distinct hospital care packages for patients with heart rhythm abnormalities

**Table S8**. Incident cases for ICD implantation for primary prevention in the Netherlands

| **Calendar year** | **Yearly total first implants primary prevention** | **Yearly total first implants** | **DBC codes** | **Declaration code** | | | | |
| --- | --- | --- | --- | --- | --- | --- | --- | --- |
|  | **65%** |  | **First ICD implants** | **33282** | **33280** | **33291** | **33294** | **33298** |
| 2012 | 2305 | 3546 | 979001112/979001114 | 1010 | 2326 | 20 | 178 | 12 |
| 2013 | 2646 | 4070 | 979001242/979001243 | 904 | 2910 | 71 |  | 185 |
| 2014 | 2756 | 4240 | 979001242/979001243 | 1073 | 3087 | 80 |  |  |
| 2015 | 2543 | 3912 | 979001242/979001243 | 1054 | 2781 | 61 |  | 16 |
| 2016 | 2543 | 3912 | 979001242/979001243 | 1080 | 2775 | 57 |  |  |
| 2017 | 2339 | 3599 | 979001242/979001243 | 1005 | 2528 | 56 |  | 10 |
| **Rounded yearly mean number** | **2500** |  |  |  |  |  |  |  |

We used the national health care database for reimbursement data (www.opendisdata.nl), managed by the Dutch Health Care Authority, and the NCDR 2015 report to determine the incident cases for ICD implantation for primary prevention in the Netherlands.

Hospital health care providers are obliged to transfer data on used health care resources to this agency and between calendar years 2012 and 2017 the national coverage was mostly 100% (except for the year 2015, 95%). ICD implantations should be discerned from (i) new implantations or replacements and (ii) performed for primary or secondary prevention. In OpenDisData new implantations can be counted by combining declaration codes 33280, 33282, 33291, 33294 and 33298 from health care product codes 979001112 and 979001114 for year 2012 and health care product codes 979001242 and 979001243 for year 2013 and beyond. See the table above for the yearly total of first implants.

The NCDR 2015 report mentioned that between 60% and 70% of first ICD implants are meant for primary prevention of SCD. If the mid-value of 65% is applied, then the yearly number of patients in the target population ranges between 2305 and 2756. The mean yearly number over these years rounded to the nearest 100 equals 2500 cases. The 2500 count of presenting cases seems acceptable for the mid-term and suitable for use in the budget impact analysis. Because of the number of historical observations was limited and the available data demonstrated a curvilinear pattern with a gradual increase followed by a gradual decline, curve-fitting models to project numbers for successive calendar years seemed either unrealistic or suggested similarly monotone outcomes like proposed here.


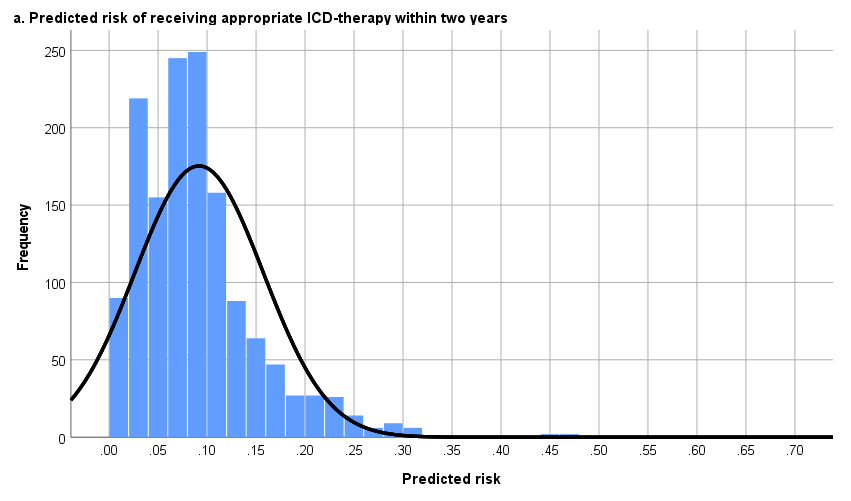


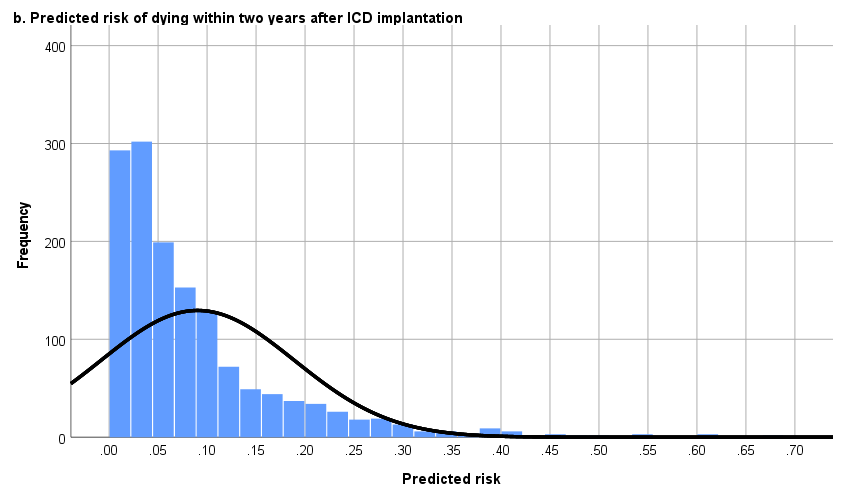


**Fig. S1a-b**. Distributions of predicted individual risks of receiving appropriate ICD-therapy (a) or dying (b) within two years following ICD implantation for primary prevention
